# Dormancy and reactivation of the seed and its microbiome: a holobiont perspective

**DOI:** 10.1128/msystems.01140-25

**Published:** 2025-12-11

**Authors:** Davide Gerna, Thomas Chadelaud, Florian Lamouche, Matthieu Barret, Armelle Darrasse, Marie Simonin

**Affiliations:** 1 Royal Botanic Gardens, Kew 41949, Wakehurst Place, United Kingdom; 2 Univ Angers, Institut Agro, INRAE, IRHS, SFR QUASAV, Angers, France; Universiteit Leiden, Leiden, the Netherlands

**Keywords:** desiccation, dormancy, holobiont, intracellular glass, microbiome, quiescence, seed, viable-but-nonculturable state

## Abstract

Desiccation-tolerant seeds provide an intriguing system for studying microbial dormancy, which includes reversible inactivation and reactivation in response to stress. Focusing on bacterial responses to desiccation and rehydration, we offer a holistic interpretation of dormancy and quiescence within the seed holobiont, highlighting both parallels and distinctions between microbes and their plant host. Based on pilot evidence, we propose that microbial dormancy supports persistence throughout the life cycle of desiccation-tolerant seeds. Transcriptomic analyses of seed-transmitted bacteria have identified genes implicated in inactivation and the viable-but-nonculturable state. Our analysis of *Xanthomonas citri* pv. *fuscans* illustrates this during seed maturation. However, the signals triggering microbial reactivation and the potential reciprocal interactions between seed dormancy and quiescence, and microbial dormancy, remain unknown. Elucidating this interplay within the seed holobiont could enhance plant growth and health either by promoting seed germination through microbial inoculation or by enabling early detection of seed-transmitted phytopathogens.

## OPINION/HYPOTHESIS

Desiccation tolerance—the ability of organisms to withstand severe water loss and subsequently revive—is a key trait acquired by seeds of most plant species during the final stages of development, when their moisture content declines to ~10% of fresh weight (1). Desiccation-tolerant seeds (hereafter seeds for simplicity) survive the removal of cellular water by accumulating protective molecules and forming intracellular glasses, which impose a metabolically inactive state called **quiescence** (*quietus*, at rest) (2) (boldface terms are defined in Box 1). Once environmental conditions become favorable, typically after rehydration and in the presence of suitable temperature, light, and oxygen, quiescent seeds resume metabolism and can germinate. However, even under these conducive hydrated conditions, seed germination may still be restricted by endogenous inhibitors (2). This seed trait, which requires additional regulatory mechanisms, is called **physiological dormancy** (*dormire*, to sleep) (Box 1).

Box 1. **Glossary**

- **Microbial dormancy**: a reversible state of reduced metabolic activity and arrested growth that enables microbes to survive under stress and resource-limited conditions. Dormant cells can undergo cycles of inactivation and reactivation, resuming full metabolism and growth either stochastically or in response to abiotic and biotic cues.
- **Seed physiological dormancy**: a type of seed dormancy resulting in the failure of viable, fully hydrated seeds to germinate under otherwise favorable conditions. Germination is actively inhibited by endogenous phytohormonal signals, driven by an imbalance between abscisic acid (inhibitor) and gibberellins (promoters). Basal metabolic and transcriptional activity persists, while germination-related pathways remain repressed until defined environmental cues (e.g., temperature, light) induce hormonal shifts that release dormancy and trigger germination.
- **Seed depth of physiological dormancy**: the degree of dormancy imposed by physiological mechanisms, such as hormonal and metabolic constraints. Among physiological dormancy types, “shallow” and “deep” dormancy differ in mechanism, responsiveness to environmental cues, and ecological role. Shallow dormancy allows more flexible germination after dispersal through faster hormonal rebalancing, whereas deep dormancy imposes more pronounced phytohormonal repression and ensures seed germination only after predictable sequences of environmental conditions (e.g., winter cold). By analogy, the terms “shallow” and “deep” may also describe microbial responsiveness to cues within the seed habitat.
- **Seed quiescence**: a reversible, externally imposed state of metabolic and growth arrest that prevents germination primarily due to the absence of sufficient water. During desiccation, cells lose water, metabolism halts, and intracellular components are stabilized through cytoplasmic vitrification (intracellular glass formation). Unlike seed physiological dormancy, quiescence does not involve endogenous physiological inhibition, and germination resumes promptly upon rehydration, provided that the seed remains viable.
- **Viable**-**but**-**nonculturable** (**VBNC**) **state**: a microbial survival strategy, primarily documented in bacteria, in which cells remain viable and retain low-level metabolic activity but lose the ability to grow on standard culture media. Typically induced by environmental stresses such as desiccation or nutrient limitation, VBNC cells maintain membrane integrity, basal gene expression, and active stress-response pathways. Reactivation requires resuscitation-promoting factors and other largely unknown cues. Although VBNC cells share with dormant cells growth arrest in response to stress, they typically display higher metabolic activity (a shallow dormancy state) and poorly characterized reactivation mechanisms. The VBNC state represents a distinct survival strategy within the broader spectrum of microbial dormancy.

As modes of suspended growth, quiescence and dormancy are not unique to seeds but are rather continuous cellular traits shared across the tree of life (2). Comparing quiescence and dormancy between seeds and their associated microbial communities (i.e., microbiomes) offers an integrated perspective on the relevance of these two traits to the survival and persistence of the seed holobiont, the functional unit comprising the plant host and its microbiomes (3).

In seeds, quiescence and dormancy are distinct physiological states. However, in microbes, these terms are often used interchangeably to describe temporarily inactive cells. In microbial populations, dormancy is defined as a reversible state of reduced metabolic activity and arrested growth under unfavorable conditions (4) (Box 1). This transition into dormancy, commonly referred to as inactivation, is followed by reactivation of metabolism and growth after stress is relieved (4). Additional microbial survival states, such as persister cells and the **viable but nonculturable** (**VBNC**) **state**, are frequently mentioned in the context of **microbial dormancy** (Box 1). Within the seed habitat, all these terms can be better understood through the concept of a microbial “dormancy spectrum,” which recognizes a continuum of growth arrest and varying **depths of dormancy** (Box 1) rather than a strict dormant-active binary (5). This spectrum also mirrors the current understanding of seed dormancy as a continuous trait expressed at the population, maternal plant, and individual seed levels (6). While this framework is described in bacteria and yeast (5), it remains less explored in fungi and archaea. Here*,* we primarily focus on non-sporulating forms of metabolic dormancy, which appear to be common and ecologically relevant across microbial communities (4, 7).

Based primarily on evidence from bacteria, we propose that the seed life cycle—from fertilization to seedling establishment—offers an intriguing model to investigate microbial dormancy transitions, including the inactivation and reactivation dynamics of the plant holobiont.

## EVIDENCE FOR MICROBIAL DORMANCY DURING SEED DEVELOPMENT

**Fig 1:**
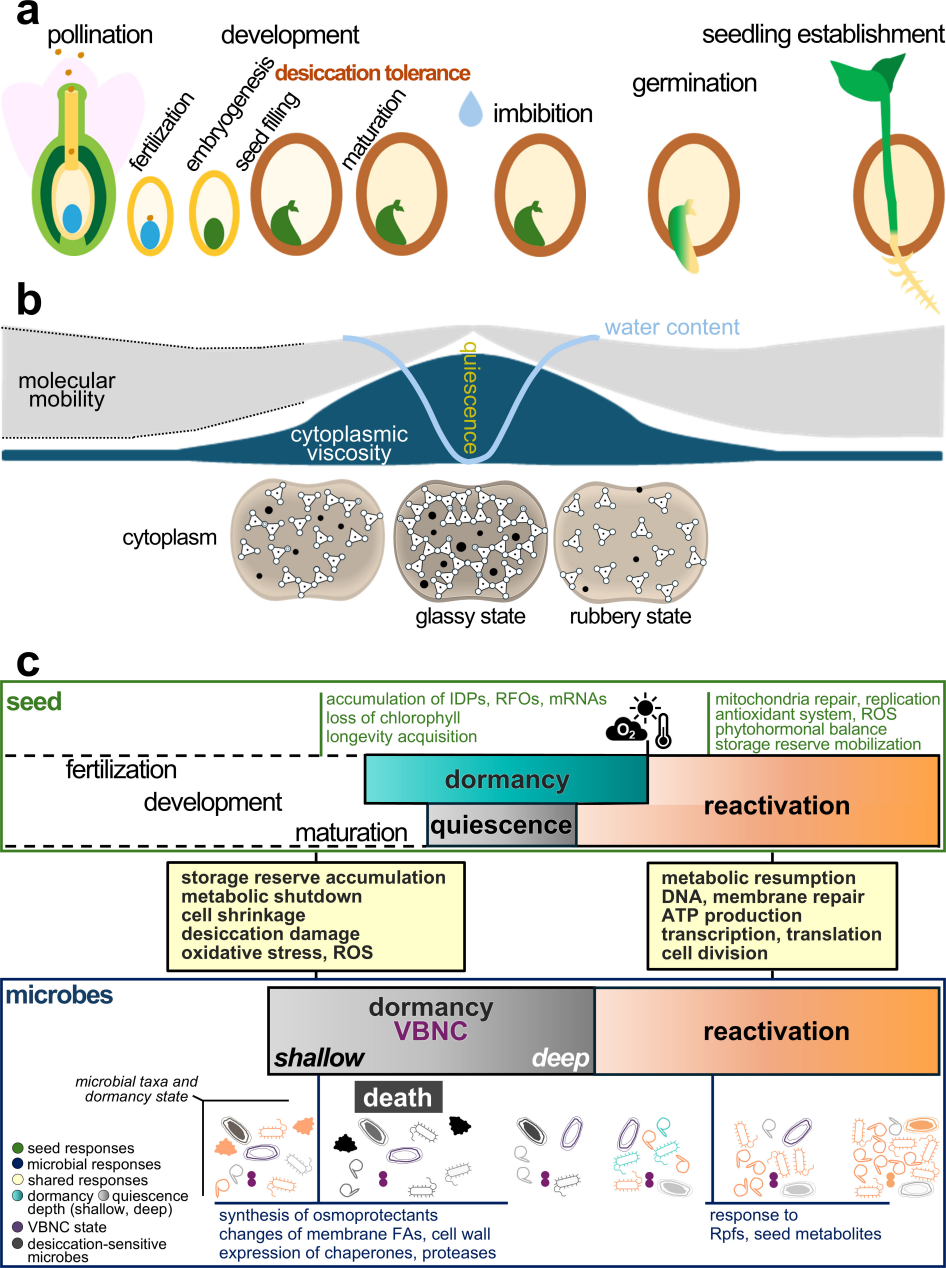
Inactivation and reactivation of the seed desiccation-tolerant holobiont. (**a**) Overview of the seed life cycle from pollination and ovule fertilization to seed development (left to right). Seed desiccation tolerance is acquired during seed filling and completed during seed maturation. The water droplet denotes metabolic reactivation upon seed imbibition, followed by germination (radicle protrusion) and seedling establishment (emergence of aerial tissues). (**b**) Schematic representation of cytoplasmic viscosity changes that regulate quiescence in desiccation-tolerant seeds. The light blue curve depicts changes in seed water content. During seed maturation, the cytoplasm of plant cells, and likely microbial cells, forms a highly viscous intracellular glass (glassy state), in which all macromolecules are densely packed, restricting molecular mobility and resulting in virtually undetectable metabolic activity. During imbibition, seed water uptake induces plasticization, relaxing the cytoplasmic matrix into a less viscous, rubbery state, with increased molecular mobility that enables metabolic reactivation and growth. (**c**) Mechanisms of inactivation and reactivation shared between seeds (green text) and microbes (blue text) are shown in yellow boxes. Color gradients reflect transitions in dormancy depth—from shallow to deep—for both the seed and its microbiome. Seed desiccation tolerance induces metabolic quiescence and, in some species, physiological dormancy. Under favorable environmental conditions, imbibition terminates seed quiescence and reactivates metabolism for seed germination. Dormant seeds require additional environmental cues (e.g., light, temperature, and oxygen), which cascade through phytohormonal and redox regulation, to break physiological dormancy before reactivating pathways of germination. Dotted lines indicate stages of the seed life cycle that are not known to directly affect seed quiescence and physiological dormancy. During seed development, seed-transmitted microbes may enter dormancy states of variable depth, including viable-but-nonculturable (VBNC) phenotypes, paralleling seed quiescence. Microbial dormancy responses may coincide with desiccation-related processes such as intracellular glass formation. The microbial shapes (bottom) represent different taxa, with colors indicating dormancy depth (from shallow to deep) or viability. This visual highlights the diversity and heterogeneity of microbial inactivation and reactivation responses to desiccation and rehydration within the seed holobiont life cycle. Upon imbibition, rehydration may also gradually reactivate microbial cells. While some microbes may resume metabolism and proliferate within hydrated seed tissues, others may remain dormant or VBNC until triggered by resuscitation-promoting factors (Rpfs) or other seed-derived signals. Abbreviations: FAs, fatty acids; IDPs, intrinsically disordered proteins such as late embryogenesis abundant proteins and dehydrins; RFOs, raffinose family oligosaccharides; ROS, reactive oxygen species.

Seed development comprises embryogenesis, seed filling with storage reserves, and seed maturation (Fig. 1a). Desiccation and the associated mechanical, osmotic, and oxidative stresses accompany seed maturation. During development, seeds that acquire the ability to survive at low water contents (desiccation tolerance) become metabolically quiescent, whereas physiological dormancy develops in only certain species. In seed-transmitted microbes, dormancy may be coordinated with seed desiccation tolerance, supporting their survival during the transition from seed maturation to germination. This coordination is documented by seed-transmitted phytopathogenic bacteria that, for instance, can survive in dry tomato seeds for several months (8). *In vitro* studies have shown that diverse seed-transmitted phytopathogenic bacteria—such as *Xanthomonas campestris* pv. *campestris* (*Xcc)* in cabbage (9), *Clavibacter michiganensis* pv. *michiganensis* in tomato (10), *Acidovorax citrulli* in watermelon (11), and *Pseudomonas syringae* pv. *syringae* in alfalfa (12)—can persist as VBNC cells and reactivate in response to environmental stress. In addition to seed-transmitted phytopathogens, shifts in the composition of culturable bacterial communities have been observed during seed development. Notably, culture enrichment from individual bean seeds followed by taxonomic profiling revealed shifts, including a decline in Enterobacterales during seed maturation (13). At this seed developmental stage, genera harboring spore formers, such as *Bacillus*, prevailed in the culturable fraction of endophytic bacteria from rice seeds (14), further supporting a role for microbial dormancy in response to seed desiccation. These patterns are consistent with the hypothesis that microbial survival strategies for desiccation, particularly dormancy, may shape microbiome succession during seed development. Therefore, we postulate that most seed-transmitted microbes persist as dormant cells during seed maturation and after dispersal in the dry state.

## DORMANCY AND INACTIVATION: MECHANISMS SHARED BETWEEN SEEDS AND MICROBES

Water availability influences the physical properties of the cytoplasm, which in turn modulate enzymatic activity and diffusion. In desiccated seeds, cellular viscosity increases through shrinkage and vitrification of the cytoplasm, which forms an amorphous matrix termed intracellular glass (Fig. 1b). Intracellular glasses impose **seed quiescence** and enable seeds to maintain metabolism at a standstill, thereby limiting deteriorative enzymatic reactions and oxidative stress (15). Thermal and mechanical analyses of seeds are used to characterize the properties of intracellular glasses, such as the glass transition temperature (15). Some of these properties have also been reported for intracellular glasses of a few microbes, including spore-forming *Bacillus* spp. and spores of the fungi *Saccharomyces cerevisiae* (unicellular) and *Talaromyces macrosporus* (filamentous) (5, 16). Although cytoplasmic vitrification remains unexplored in seed-transmitted microbes, we envisage it as a conserved mechanism to survive desiccation and persist in the dry state of the seed holobiont.

Transcriptome studies have revealed an intricate interplay of mechanisms enabling microbial desiccation tolerance. During desiccation, bacteria and yeast activate stress-responsive pathways, including the synthesis of trehalose and other osmoprotectants, enrichment of saturated fatty acids in membranes (17, 18), and upregulation of genes encoding efflux pumps, reactive oxygen species (ROS) scavengers, and the DNA repair machinery, which collectively protect cells from oxidative and structural damages (17–19). These responses are accompanied by the downregulation of energy-intensive processes such as DNA replication, transcription, motility, secretion, and cell division in bacteria (17, 18). At very low cellular water contents, some taxa sporulate as an extreme survival strategy (17). Reversible osmotic and oxidative stress accompanying desiccation can trigger bacteria to enter the VBNC state by involving dynamic physiological changes and upregulation of genes for DNA replication and repair, chaperone expression, cell wall remodeling, and modulation of carbon metabolism through respiratory pathways (9, 12, 20–23). Notably, dormant and VBNC cells share overlapping transcriptomic responses to desiccation, including suppression of central carbon metabolism and sugar transport, respiratory shifts, adjustment of membrane fatty acids, and halted cell division (20–22). These responses illustrate conserved bacterial survival strategies operating at different depths along the dormancy spectrum (5), which may also occur during the seed life cycle, when bacteria experience desiccation-related stresses (Fig. 1c). In contrast, transcriptomic changes during desiccation of other seed-transmitted microbes remain less well characterized. Filamentous fungi and archaea may rely on distinct or as-yet-unknown mechanisms beyond sporulation to persist in the desiccated seed.

**Fig 2:**
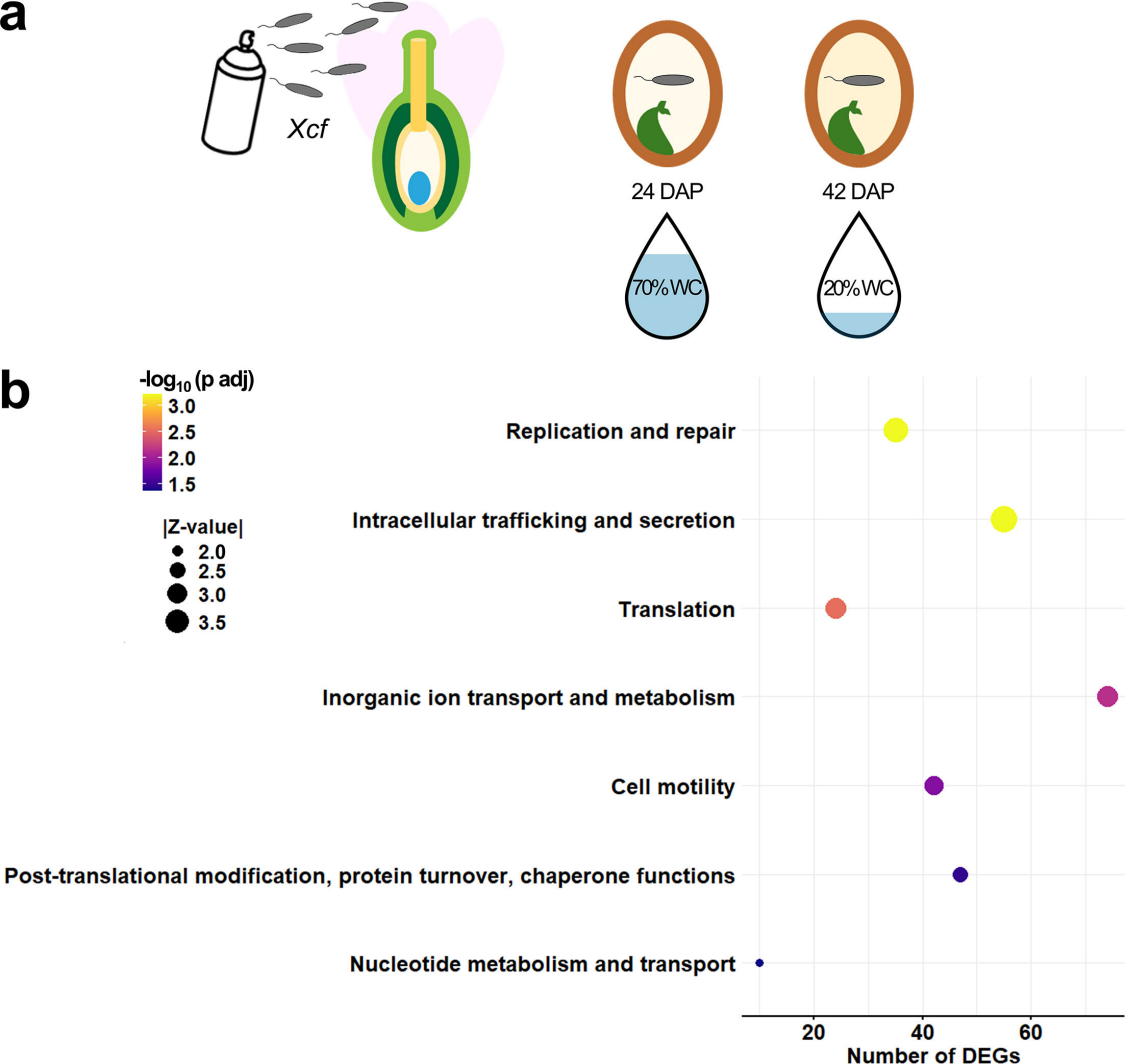
Bacterial preparation for dormancy during seed maturation. Transmission assay of *Xanthomonas citri* pv. *fuscans* (*Xcf* ) to seeds of common bean. (**a**) Flower buds and open flowers were spray-inoculated with a water-based suspension of *Xcf* at 10^6^ colony-forming units mL^−1^. Seeds were harvested at early (24 days after pollination: DAP, ~70% seed water content: WC) and late maturation (42 DAP, ~20% seed WC), and *Xcf* RNA was extracted and sequenced after enrichment (24). (**b**) RNA-seq analysis of raw mapped *Xcf* reads was performed using DESeq2 (25) to determine differentially expressed genes (DEGs) between late and early seed maturation at an adjusted *P*-value (*P* adj) < 0.05. Cluster of orthologous groups (COGs) was conducted with COG annotation of *Xcf* DEGs with eggNOG-mapper v2 (https://github.com/eggnogdb/eggnog-mapper/tree/master/eggnogmapper), and COG enrichment was assessed using Fisher’s exact test (adjusted *P*-value < 0.05). Enrichment scores (Z-values) and the corresponding adjusted *P*-values for each COG category, both obtained from Fisher’s exact test, are displayed. Raw mapped RNA-seq reads of *Xcf* during seed development are from (24).

Our analysis of RNA-seq data from *Xanthomonas citri* pv. *fuscans* (*Xcf*), sourced from a study on common bean seed development (24), served as a proof-of-concept for microbial inactivation responses within the seed system (Fig. 2a), but validation across other seed-associated microbes is needed. Comparative transcriptomics between early and late seed maturation (~70% and ~20% seed water content, respectively) revealed seven functionally enriched clusters of orthologous groups in *Xcf* cells (Fig. 2b). These groups encompassed genetic fidelity and replication (DNA repair and nucleotide metabolism), protein homeostasis (translation, post-translational modification, and chaperone functions), structural dynamics (cell motility and secretion systems), and metabolic adaptation (inorganic ion transport) (Fig. 2b). In particular, changes in the expression of genes coding for stress-responsive chaperones, DNA repair, and motility apparatus align with previous studies of bacterial desiccation (18). Notably, the *Xcf* transcriptional profile *in planta* mirrored bacterial desiccation survival responses and signatures of entry into the VBNC state observed *in vitro*, suggesting that microbial cells across the dormancy spectrum, with restricted metabolism, may exhibit similar desiccation-responsive transcriptional profiles.

Whereas microbes adopt metabolically restricted states, such as dormancy (26), to survive desiccation, physiological dormancy of the host seed is regulated by endogenous networks that sense environmental cues to resume growth upon rehydration (6). **Seed physiological dormancy** is primarily maintained by the phytohormone abscisic acid, which suppresses growth and delays germination (6). This effect is modulated by antagonistic interactions with growth-promoting phytohormones, such as gibberellins and cytokinins (6). Regulation of seed physiological dormancy is further fine-tuned by redox signaling and epigenetic modifications, which influence hormone sensitivity and the expression of dormancy-related genes. For instance, oxidative changes affect the release of physiological dormancy during dry seed storage (27), and adjustments of the cellular redox state are also relevant to seed germination (28). While these processes are documented in seeds, the influence of seed-transmitted microbes and their metabolites on seed phytohormonal and redox balance remains largely unexplored. Additionally, cytoplasmic vitrification during seed maturation leads to seed quiescence and may similarly restrict the activity of seed-associated microbes, contributing to microbial dormancy. Thus, while seed quiescence results from biophysical constraints, seed physiological dormancy is actively maintained by phytohormonal regulation of endogenous metabolism (Box 1).

In summary (Fig. 1c), current evidence suggests that plant cells and seed-transmitted bacteria share several cellular mechanisms to halt metabolic activity and growth during seed maturation, described as seed quiescence and microbial dormancy.

## DORMANCY AND REACTIVATION: MECHANISMS SHARED BETWEEN SEEDS AND MICROBES

Similar to inactivation, the desiccation-tolerant partners of the seed holobiont exhibit common structural and molecular mechanisms of reactivation. With initial seed water uptake (i.e., imbibition), plant intracellular glasses relax into more fluid matrices (the so-called rubbery state) with increased molecular mobility conducive to enzymatic activity and long-distance diffusion (Fig. 1b)—see also (15). Also in microbes, the association between cytoplasmic relaxation (i.e., devitrification) and metabolic resumption has found support (16, 29, 30).

Repairing desiccation damage is a priority for reawakening the seed holobiont. Membranes and DNA, key cellular components, are among the first structures to be repaired when a seed and its microbes reactivate (Fig. 1c). The seed promptly initiates mitochondrial repair, differentiation, and replication, while enzymes modulate ROS concentrations and regenerate non-enzymatic antioxidants (31). During imbibition, seed-stored mRNAs accumulated during late maturation assemble into polysomes to support timely protein synthesis, including for enzymes involved in macromolecular repair and phytohormonal regulation, even prior to substantial *de novo* transcription (Fig. 1c)—see also (32). Before mitochondria return to full functionality, germinative metabolism relies on glycolysis and the pentose phosphate pathway (PPP). Under low oxygen, alcoholic fermentation and the Perl’s pathway ensure a continued energy supply. With active mitochondria, aerobic respiration and the PPP become the predominant central metabolic pathways fueling seed germination (31). Notably, the production of ATP is also an early signature of microbial reactivation after dormancy (5, 29), and *de novo* transcription and translation are other conserved mechanisms of reactivation of the seed holobiont. However, reactivation may not be tightly coordinated between the seed host and its microbiome, and different microbial taxa may reactivate at distinct stages following imbibition (Fig. 1c).

Upon imbibition, viable seeds resume basal metabolism, ending quiescence and potentially providing water and nutrients that enable some dormant microbial cells to reactivate. Although direct evidence for microbial reactivation prior to seed-dormancy break is limited, seeds initiate defense mechanisms, such as the release of exudates, to restrict microbial growth during imbibition. This interplay likely helps maintain a balance between microbial activity and seed defense until the seed releases physiological dormancy (33).

In bacteria, reactivation from the VBNC state involves resuscitation-promoting factors (Rpfs) (34) and quorum-sensing signals (35). We hypothesize that reactivation of seed-transmitted microbes is triggered by plant-derived signals, potentially including some primary metabolites mobilized shortly after imbibition (36, 37).

Compared to inactivation, more direct evidence is available for microbial reactivation from dormancy following seed metabolic resumption (38). Nonetheless, the identity of the signal (i.e., single or multiple metabolites of the seed endosphere, seed exudates, or simply water) essential to reactivate microbes from dormancy and the VBNC state remains an open question.

## HARNESSING MICROBIAL DORMANCY IN THE SEED HOLOBIONT FOR IMPROVED PHYTOPATHOGEN DETECTION AND MICROBIOME ENGINEERING

Unlocking the triggers of microbial dormancy and the VBNC state in the seed holobiont holds promise for improving phytopathogen detection and microbiome engineering. While some phytopathogens, including fungi, rely on sporulation—a specialized survival strategy at one extreme of the microbial dormancy spectrum (5)—many seed-transmitted bacteria, especially non-sporulating forms, remain difficult to detect because they enter dormancy during seed maturation and storage. Understanding the mechanisms that control reactivation, particularly of non-sporulating isolates, could help synchronize microbial activity with seed germination, enhancing plant health and the efficacy of beneficial inoculants.Cultured

However, practical limitations remain. Microbial dormancy and the VBNC state likely contribute to false negatives in culture-based seed-health tests to detect phytopathogens. Molecular methods such as PCR are often preferred because they do not require culturing, but they may amplify relic DNA, particularly in disinfected seed lots, thereby reducing accuracy (39). Seed germination assays to increase microbial load and monitor disease symptoms in young seedlings (i.e., grow-out tests) offer an alternative to conventional culture-based detection by stimulating microbial reactivation in the host and allowing symptom-based detection of phytopathogens. For instance, *C. michiganensis* pv. *michiganensis* has been shown to exit the VBNC state in tomato seedlings (10). However, VBNC phytopathogenic bacteria often reactivate at population sizes too low to cause visible symptoms (10, 11). To enhance sensitivity and tackle this limitation, quantitative PCR (qPCR) assays combined with growth-out tests that enrich the reactivated populations of VBNC cells (the “seed-qPCR” approach) have been successfully tested for *Xcc* in *Brassica oleracea* seeds (40). Additionally, culturing can be improved using media that mimic the seed-endosphere metabolite profile, enabling isolation of rare taxa (36, 37). The differential response of bacterial taxa to Rpfs offers another avenue for innovative detection methods. Indeed, Rpfs could be engineered to selectively reactivate dormant or VBNC phytopathogens (41). Besides harmful microbes, timely reactivation of beneficial seed-transmitted endophytes may strengthen plant defenses against biotic and abiotic stresses (42), opening new possibilities for microbiome-informed plant management.

Dormancy of the seed holobiont could also be leveraged through microbial inoculations, such as spraying the floral stigma with suspensions of growth-promoting microbes, which can colonize developing seeds and inactivate during seed maturation (43). In the seed holobiont life cycle, microbial inoculations appear to modulate multiple plant traits, from fruit set rates to phytopathogen transmission, germination, seedling establishment, and hence plant productivity (24, 43). To optimize the inoculation efficiency of both individual strains and consortia, microbial selection could be informed by microbial dormancy traits, such as desiccation tolerance, that reflect adaptation to the seed niche, survival through seed development, and prompt reactivation upon imbibition.

In summary, advancing knowledge of microbial dormancy in the seed holobiont promises innovative strategies to improve phytopathogen detection and guide microbial inoculation approaches (e.g., flower inoculation) that promote microbial persistence and timely activation. Synchronizing microbial reactivation with the seed’s exit from quiescence and physiological dormancy may enhance the contribution of microbes to sustainable plant production.

## SEED HOLOBIONT DORMANCY: WHAT IS NEXT?

This opinion highlights shared mechanisms and possible interplays between the physiological dormancy of desiccation-tolerant seeds and their associated microbes, focusing on bacterial communities. We propose the seed holobiont as a valuable model for interkingdom studies on suspended life and reactivation, with potential implications for host-microbe co-survival, fitness, and evolutionary trajectories. Such a framework could also complement studies of dormancy in other multipartite biological systems, including filamentous fungi, desiccation-sensitive seeds, and seeds with contrasting morphologies or chemical compositions. Building on the conceptual framework presented here, we outline directions to guide future research on dormancy within the seed holobiont.

- Although seeds and their microbes share some survival strategies, the conservation or divergence of dormancy mechanisms across seed-associated microbial taxa at the biochemical, molecular, and (epi)genetic levels remains poorly characterized. In particular, desiccation tolerance appears key for inducing dormancy in seed-transmitted bacteria. The physical properties of the intracellular glasses formed during desiccation of seed-transmitted microbes have not been measured and may help explain differences in the persistence and reactivation dynamics among microbial taxa across the seed life cycle.
- In seeds, the signals triggering microbial reactivation from dormancy and the VBNC state are far from being resolved. Determining which seed-transmitted microbes reactivate with imbibition alone, or which require certain plant metabolites, phytohormones, and redox cues, represents another important research avenue.
- The extent to which seed-transmitted microbes shape the host’s **depth of physiological dormancy** and its release during germination, and conversely how seed physiological dormancy affects microbial activity, remains largely unexplored. In particular, the impact of microbial metabolism on seed and seedling hormonal and redox balance warrants detailed study.
- Seed holobiont dormancy has ecological and evolutionary significance. It may influence plant persistence in seed banks, adaptation, and the biogeography of microbiomes through seed dispersal, highlighting an additional area for investigations across plant species.

In conclusion, advancing our understanding of dormancy and reactivation within the seed holobiont requires multidisciplinary efforts, which could reveal interkingdom survival strategies and unlock novel opportunities to enhance plant growth, improve resilience to biotic and abiotic stresses, and conserve biodiversity.
